# Detection of tumor-derived extracellular vesicles in plasma from patients with solid cancer

**DOI:** 10.1186/s12885-021-08007-z

**Published:** 2021-03-24

**Authors:** Silvia R. Vitale, Jean A. Helmijr, Marjolein Gerritsen, Hicret Coban, Lisanne F. van Dessel, Nick Beije, Michelle van der Vlugt-Daane, Paolo Vigneri, Anieta M. Sieuwerts, Natasja Dits, Martin E. van Royen, Guido Jenster, Stefan Sleijfer, Martijn Lolkema, John W. M. Martens, Maurice P. H. M. Jansen

**Affiliations:** 1 Department of Medical Oncology, Erasmus MC Cancer Institute, Erasmus MC, Room Be400, Dr. Molewaterplein 40, 3015 GD Rotterdam, The Netherlands; 2 Department of Clinical and Experimental Medicine - Center for Experimental Oncology and Hematology, University of Catania, Catania, Italy; 3 Department of Urology, Erasmus MC Cancer Institute, Erasmus MC, Rotterdam, The Netherlands; 4 Department of Pathology, Erasmus MC Cancer Institute, Erasmus MC, Rotterdam, The Netherlands; 5 Department of Cancer Genomics Netherlands, Erasmus MC Cancer Institute, Erasmus MC, Rotterdam, The Netherlands

**Keywords:** EV-RNA, cfDNA, Liquid biopsy, dPCR

## Abstract

**Background:**

Extracellular vesicles (EVs) are actively secreted by cells into body fluids and contain nucleic acids of the cells they originate from. The goal of this study was to detect circulating tumor-derived EVs (ctEVs) by mutant mRNA transcripts (EV-RNA) in plasma of patients with solid cancers and compare the occurrence of ctEVs with circulating tumor DNA (ctDNA) in cell-free DNA (cfDNA).

**Methods:**

For this purpose, blood from 20 patients and 15 healthy blood donors (HBDs) was collected in different preservation tubes (EDTA, BCT, CellSave) and processed into plasma within 24 h from venipuncture. EVs were isolated with the ExoEasy protocol from this plasma and from conditioned medium of 6 cancer cell lines and characterized according to MISEV2018-guidelines. RNA from EVs was isolated with the ExoRNeasy protocol and evaluated for transcript expression levels of 96 genes by RT-qPCR and genotyped by digital PCR.

**Results:**

Our workflow applied on cell lines revealed a high concordance between cellular mRNA and EV-RNA in expression levels as well as variant allele frequencies for *PIK3CA*, *KRAS* and *BRAF*. Plasma CD9-positive EV and GAPDH EV-RNA levels were significantly different between the preservation tubes. The workflow detected only ctEVs with mutant transcripts in plasma of patients with high amounts (> 20%) of circulating tumor DNA (ctDNA). Expression profiling showed that the EVs from patients resemble healthy donors more than tumor cell lines supporting that most EVs are derived from healthy tissue.

**Conclusions:**

We provide a workflow for ctEV detection by spin column-based generic isolation of EVs and PCR-based measurement of gene expression and mutant transcripts in EV-RNA derived from cancer patients’ blood plasma. This workflow, however, detected tumor-specific mutations in blood less often in EV-RNA than in cfDNA.

**Supplementary Information:**

The online version contains supplementary material available at 10.1186/s12885-021-08007-z.

## Background

Many cell types, including cancer cells [1], release extracellular vesicles (EVs) in various body fluids [2–6]. EVs (size range 40-5000 nm) are formed by: 1) Vesicle budding from the cellular membrane (microvesicles), 2) apoptosis (apoptotic bodies) and 3) via the endocytic or the secretory pathway (multi-vesicular bodies) [3, 7–10]. Recent studies showed that EVs are released into the circulation during various pathological processes, including cancer. Circulating tumor-derived EVs (ctEVs) are a small portion of EVs originating from tumor cells which carry their cargo to neighboring cells or distant organs [7, 11, 12]. EVs have heterogeneous membrane compositions and contents [1, 13] and their counts increase over time in blood during disease progression [14]. Most research on EVs was focused on proteomics [15, 16]. EVs contain intact and fragmented mRNA [1, 6, 14], miRNA [7, 17–19], small and long non-coding RNA (ncRNAs), but also tRNAs, and rRNAs [7, 17, 18, 20–22]. Where freely circulating mRNA is prone to rapid degradation outside cells, it is hypothesized that mRNA molecules remain stable within vesicles. Current data favor the hypothesis that mRNAs present in cells do not end up in EVs at random but that only specific mRNA molecules are selectively packaged inside these vesicles [7, 14]. However, before RNA derived from EVs (EV-RNA) can be used as biomarkers for disease detection and for the prediction of prognosis or therapy response in cancer [22], development of reliable detection methods is urgently required. The purpose of this study was to establish such a pipeline to detect ctEVs by establishing a workflow for the isolation and characterization of EVs and EV-RNA. This workflow was firstly tested in cell line models and subsequently applied to analyze EV-RNA isolated from plasma of 20 patients with metastatic cancer. Additionally, we addressed whether our reported pre-analytical conditions established for plasma cfDNA analyses [23] were suitable for isolation and analysis of EVs. Finally, we used this workflow to detect ctEVs by mutant transcripts and evaluate gene expression in both cell line and patient-derived EV-RNA.

## Methods

### Study design

**Fig. 1:**
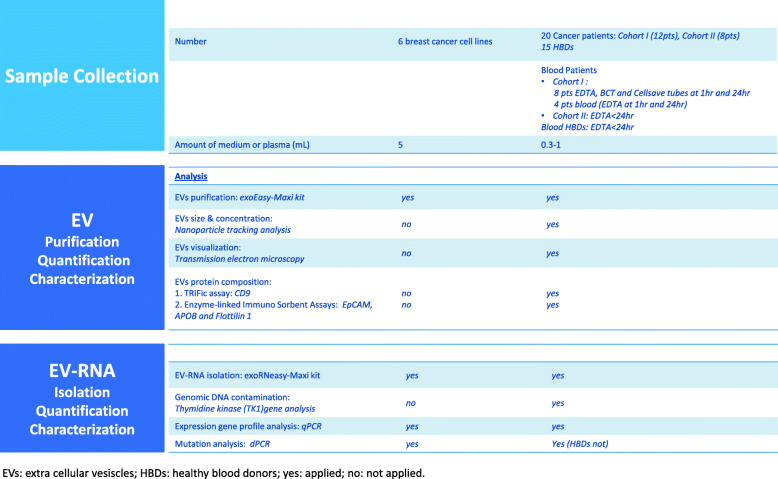
Flow chart of processing samples. EVs: extra cellular vesiscles; HBDs: healthy blood donors; yes: applied; no: not applied

Figure 1 shows a detailed overview of the study design.

### Cell culture

In this study we used 6 human breast cancer cell lines from the American Tissue Cell Culture (ATCC), i.e. BT20, MDA-MB-231, MDA-MB-361, MDA-MB-435s, MCF7, T47D. Cell lines were cultured at 37 °C in humidified air with 5% CO_2_ in RPMI 1640 medium supplemented with 10% fetal bovine serum (FBS), 100 U/mL penicillin, 100 μg/mL streptomycin and 50 μg/mL gentamycin. After cell lines reached 80% confluency, cells were washed with PBS and cultured in RPMI 1640 medium without the addition of FBS (conditioned medium) for 48 h. Then cells and conditioned medium were collected. The cells were used for DNA and RNA extraction and the conditioned medium was used for Extracellular Vesicles (EV) and EV-RNA purification. Additionally, cell line authenticity was determined by comparing Short Tandem Repeat (STR) analysis using the Powerplex 16 system (Promega, Cat. No: DC6531) with STR marker references from the American Type Culture Collection (ATCC).

### Sample characteristics and plasma collection

A total of 20 patients with various types of cancer and documented metastases were included in the study, who donated blood within the Erasmus MC Cancer Institute, Rotterdam, Netherlands. Blood was donated in 2015–2016 for Cohort I (12 patients) and in 2017 for cohort II (8 patients). None of the patients received systemic treatment at time of blood draw. Blood from 15 healthy blood donors (HBDs), collected between September 2016 and September 2017, were provided by the Sanquin Blood Bank South-West Region (The Netherlands). The study was approved by the institutional review board of the Erasmus MC Medical Ethical Committee (Erasmus MC ID: MEC-15-616) and conducted in accordance with the Declaration of Helsinki and applicable regulatory requirements. The study was carried out according to the REMARK guidelines and Code of Conduct of the Federation of Medical Scientific Societies in the Netherlands (https://www.federa.org/codes-conduct). All patients provided written informed consent before blood collection and data analysis. The characteristics of the patients are summarized in Table 1. All patients had a known somatic variant in their primary tumor or metastatic lesion, which was also detected in plasma cfDNA from 9/12 patients (Cohort I) and all 8 patients (Cohort II).

**Table 1:** Characteristics of the clinical samples included in this study

				*EV-RNA* *isolation*		*TRIFic CD9* *Analysis*	*Target gene* *analysis*		*Mutation* *Analysis*	*Variant Allele* *Frequency*		
	#	Primary tumor	Mutation in primary tumor	Plasma Input (mL)	Yield^a^ (ng)	CD9-EV Europium^a^ (count × 10^**5**^)	GAPDH^a^ (cp × 10^**4**^)	Target gene^a^ (cp × 10^**2**^)	MT|WT cp^a^ in EV-RNA	EV-RNA (%)	Primary Tumor (%)	cfDNA^c^ (%)
*Cohort I*	*1*	**Cholangio** **Carcinoma**	*KRAS* G12D (c.35G > A)	0.8	646	1.1	2.8	7.8	0|600	0	40	0
	*2*	**Colorectal** **Carcinoma**	*PIK3CA* H1047R (c.3140A > G)	0.7	908	3.7	0.2	0	0|0	0	38	2.7
	*3*	**Breast Cancer**	*PIK3CA* H1047L (c.3140A > T)	0.8	301	2.9	4.1	6.7	0|432	0	26	0
	*4*	**Melanoma**	*BRAF* V600E (c.1799 T > A)	0.8	1073	1.9	1.6	2.1	0|395	0	3	1
	*5*^b^	**Colorectal** **Carcinoma**	*KRAS* G13D (c.38G > A)	1.3	372	8.4	5.3	10	202|1161	15	60	64.5
	*6*	**Colorectal** **Carcinoma**	*KRAS* G12D (c.35G > A)	0.8	250	2.5	0.9	3.1	0|315	0	44	7.3
	*8*	**Melanoma**	*BRAF* V600E (c.1799 T > A)	0.7	820	4.9	6.4	8.8	0|475	0	64	35.7
	*9*	**Melanoma**	*BRAF* V600E (c.1799 T > A)	0.65	205	1.5	2.2	2.6	0|373	0	70	4.2
	*13*	**NSCLC**	*EGFR* T790M (c.2369C > T)	0.8	473	1.2	1	0.2	0|0	0	17	0.9
	*14*	**Melanoma**	*BRAF* V600E (c.1799 T > A)	0.8	655	0.8	1.5	1.9	0|410	0	56	5.8
	*15*^b^	**NSCLC**	*EGFR* T790M (c.2369C > T)	1.05	368	4.1	2.7	3	288|938	24	65	26.3
	*16*	**Melanoma**	*BRAF* V600E (c.1799 T > A)	0.8	137	1.2	5.3	6.6	0|756	0	50	0
*Cohort II*	*17*	**NSCLC**	*KRAS* G12C (c.34G > T)	0.9	130	5.6	1.9	5.9	0|673	0	32	0.5
	*18*	**Melanoma**	*BRAF* V600E (c.1799_1800delinsAA)	0.9	154	3.3	1.2	1.3	0|184	0	50	4.4
	*19*	**Melanoma**	*BRAF* V600K (c.1798_1799delGTinsAA)	0.6	174	4.3	1.8	3.1	0|556	0	38	2.6
	*20*	**Colon** **Carcinoma**	*KRAS* G12D (c.35G > A)	0.9	414	3.1	3.6	1.1	0|2322	0	45	4.5
	*21*^b^	**Colon** **Carcinoma**	*KRAS* G13D (c.38G > A)	0.9	487	4.8	3.6	1.1	34|1521	2.2	40	23.8
	*22*	**Rectum-carcinoma**	*KRAS* G12V (c.35G > T)	1	202	4.4	1.3	3.5	0|511	0	UNK	0.9
	*23*	**Colon** **Carcinoma**	*KRAS* G13D (c.38G > A)	1	154	9.6	3.0	7.7	0|949	0	50	2.3
	*24*	**Melanoma**	*BRAF* V600K (c.1798_1799delGTinsAA)	0.9	294	3.3	5.0	8.6	0|786	0	55	0.9

^a^ per mL plasma

^b^ Mutant EV-RNA transcripts detected

^c^ cfDNA analyses have previously been performed and described (van Dessel, L. F. et al. Mol Oncol, doi:10.1002/1878-0261.12037 (2017)

*VAF* Variant Allele Frequency, *EV* Extracellular Vesicle(s), NSCLC: Non-Small Cell Lung Cancer, *UNK* Unknown, *MT* Mutant, *WT* Wild-type, cp: copies

The blood collection methods for Cohort I were previously described [23]. Briefly, blood was collected in three types of vacutainer tubes (EDTA, Cell-Free DNA BCT, CellSave) and processed into plasma at 1 h, and 24 h after blood draw. Plasma was obtained from the blood after centrifugation at 1711 g for 10 min followed by 12,000 g for 10 min, both at room temperature. From the 16 cases of the previous study [23], only 12 patients were eligible in the current study for detection of mutant transcripts in EV-RNA with digital PCR (dPCR) mutation assays. Plasma (range 0.3–1.0 mL) processed at 1 h and 24 h after blood draw was evaluated for all 12 patients when collected in EDTA and for 8 patients when collected in BCT and CellSave tubes. For 15 HBDs and Cohort II of 8 metastatic cancer patients, blood was collected in EDTA tubes and processed into plasma within 12–24 h (< 24 h) after venipuncture, by using the above-described protocol. Then, plasma was stored at -80 °C in 1 mL aliquots until further processing.

### Isolation of extracellular vesicles

The exoEasy-Maxi kit (Qiagen, Cat. No: 77064) was used according to the manufacturer’s protocol to isolate EVs from 5 mL of conditioned culture medium and 0.3–1.0 mL of plasma. Briefly, medium and plasma were filtered using a 0.8 μm syringe filter (Millipore, Cat. No: SLAA033SS) to remove larger particles such as apoptotic bodies and cell fragments, followed by purification of EVs by mixing 1 volume of sample (filtered medium or plasma) with 1 volume of XBP buffer. Subsequently, this mix was added to an ExoEasy spin column, centrifuged for 1 min at 500 g and 4 °C. The membrane bound EVs were obtained by eluting with 400 μL XE buffer and centrifugation for 5 min at 500 g and 4 °C. The isolated EVs from HBDs and patients plasma were subsequently quantified and characterized following MISEV2018 guidelines [24]. For this, EVs were evaluated by nanoparticle tracking analysis, immuno-assays and transmission electron microscopy.

### Nanoparticle tracking analysis of extracellular vesicles

The sizes and concentration of extracellular vesicles were evaluated by NTA using the NanoSight NS300 (Malvern Panalytical, Malvern, UK), with a blue laser 488 nm and sCMOS camera. EV pellets from plasma were diluted 1:1000 (v/v) with Phosphate Buffered Saline (PBS). Each sample was recorded and analyzed for one minute in five replicate measurements by NTA 3.0 software to determine particle concentration and sizes.

### Transmission electron microscopy (TEM)

EVs purified from plasma were evaluated by TEM. Plasma EV-pellets were diluted in 10 μL PBS and 10 μL of this was added on to a formvar/carbon-coated 400 mesh copper grid for 7 min. Grid staining was performed with Uranyless EM Stain for 1 min (negative stain). Grids were air-dried, and visualized with an TALOS L120C TEM at 120 kV at 11 k–45 k magnification.

### Protein content characterization of extracellular vesicles

The EVs from plasma were characterized for (non-)tissue specific transmembrane protein CD9, and EpCAM (MISEV2018 Category 1), cytosolic protein FLOT1 (Category 2), and non-EV co-isolated structures by apolipoprotein APOB (Category 3). These analyses were performed by Europium Time-Resolved Immunofluorescence assays (TRIFic) assay for CD9 or by Enzyme-linked Immuno Sorbent Assays (ELISA) for EpCAM, APOB and FLOT1.

The protein content of EVs were evaluated with ELISA assays for EpCAM (Abcam, cat# ab264632), APOB (Abcam, cat# ab108807), and FLOT1 (Aviva System Biology, cat# OKEH02189). The ELISA assays were performed according to the protocols of manufacturers using with 50–100 μL of isolated EVs purified with the exoEasy-Maxi kit. In Short, provided standards were diluted as instructed and 100 μL of each dilution was pipetted in the provided 96 wells microtiter plate. If less than 100 μL of sample was used, the volume was brought up to 100 μL with XE-buffer (ExoEasy elution buffer) for EV preparations or PBS for plasma samples. Both XE-buffer and PBS were used as background signal controls. To prevent inter experiment variations all samples were analyzed on the same microtiter plate and time for each Elisa. All ELISA incubation and washing steps were performed with gentle shaking of solutions. Absorbances were measured at 450 nm with a microplate reader (version 5.2, Bio-Rad) software and OD values were corrected for background signal. The generated standard curves were used to calculate the protein concentrations. Additionally, all protein concentrations were normalized based on the plasma volume used for EV purification.

In Contrast to the ELISA analysis, The EV marker CD9 was directly analyzed in plasma specimen or conditioned culture medium using TRIFic exosome assays (CD9: Cat. No.: EX101, Cell Guidance Systems) following the manufactures guidelines and as previously reported [25]. Briefly, streptavidin-coated plates were incubated with biotinylated CD9 for 1 h at room temperature. Supernatant was removed and plates were washed with buffer (Kaivogen Oy, Turku, Finland, Cat. No: 42–01) in an automated plate washer (TECAN Columbus). Subsequently, 10 μL of filtered plasma or culture medium and 90 μL PBS were transferred to the wells, incubated for 1 h and washed and incubated with 100 μL Europium-labeled CD9 antibodies for 1 h, all at room temperature. After another wash step, 100 μL enhancement solution was added and incubated for 15 min at room temperature. Europium time-resolved fluorescence was subsequently measured at 615 nm wavelength by a Wallace Victor2 fluorometer (Perkin Elmer, Cat. No: 1420–020).

### Cellular and EV-RNA isolation

RNA was extracted from cells collected after incubation on conditioned medium using the RNeasy mini kit (Qiagen, Cat. No: 74104) according to the manufacturer’s protocol. Briefly, after filtering the medium or plasma and binding EVs to the exoEasy spin Column, EVs bound to the silica membrane were lysed using 700 μL of Qiazol (Qiagen, Cat. No: 79306) and the QIAzol RNA mix was collected by centrifugation for 5 min at 4 °C and 500 g. Then samples were thoroughly mixed with chloroform and followed by centrifugation at 12,000 g and 4 °C. After multiple washing steps, the purified (EV-)RNA was eluted in 20 μL RNase-free water and quantified with the Thermo Scientific NanoDrop 2000 Spectrophotometer.

### DNase treatment and cDNA generation

Prior to cDNA generation, 50–400 ng (EV)-RNA was pretreated with 1 Unit RNase-free DNase I (New England Biolabs, Cat. No: M0303S) at 37 °C for 10 min to remove contaminating DNA. DNase was inactivated by 1 μL EDTA (50 mM) at 75 °C for 10 min. The resulting 10 μL of DNase treated sample was used to generate cDNA with the SuperScript VILO cDNA Synthesis Kit (Thermo Fisher, Cat. No: 11754250) according to the manufacturer’s protocol. After cDNA generation, samples were treated with 2 Units of Ambion RNase H (Thermo Fisher, Cat. No: AM2293) and incubated at 37 °C for 30 min to remove any residual RNA.

A 136 bp fragment located in intron 2 of the Thymidine kinase (*TK1*) gene was analyzed in our ER-RNA samples after DNase treatment and cDNA generation to demonstrate successful removal of DNA contamination, using the following primers and probes:

Forward primer: 5′-CTCTGGGAACAACTCTGGGATGAGG-3′; Reverse primer: 5′-ACTCAGGTGGTCCCAGGAAGTGTGG-3′ and labeled MGB probe sequence: 5′-FAM-GAAGGCAG-3′. The analysis was performed on the Quant 3D Studio digital PCR system (see below).

### Gene expression profile analysis

Simultaneous expression analysis of 96 genes previously reported by us [26, 27] was performed in duplicate on total cellular RNA and on EV-RNA from 5 cell lines, and on EV-RNA of 6 patients. The selected genes were more abundant expressed in tumor cells than in white blood cells [27]. After cDNA generation, linear multiplex pre-amplification was applied using 96 target specific Taqman assays and TaqMan PreAmp according to the manufacturer’s instructions. Next, pre-amplified cDNA preparations were analyzed in a Mx3000P Real-Time PCR System (Agilent, Amsterdam, The Netherlands), with individual TaqMan Gene Expression Assays and TaqMan Universal PCR Master Mix, no AmpErase UNG (Thermo Fisher, Cat. No: 4324018). Levels of the reference genes *HMBS*, *HPRT1* and *GUSB* were used to control sample loading and RNA quality [26, 27]. Gene expression levels were quantified using the delta quantification cycle threshold (ΔCt) method, i.e. is the difference between the average Ct of the reference genes minus the Ct of the target gene. EV-RNA expression profiles from cell lines were compared with their cellular mRNA expression profiles to characterize genes with enriched expression in EVs. Patient plasma EV-RNA was also evaluated and was from plasma without circulating tumor DNA (ctDNA) nor circulating tumor EVs (ctEVs) (P1, P3), plasma with only ctDNA (P6, P13) and plasma with both ctDNA and ctEVs (P5, P15). Additionally, the EV-RNA expression profiles from 6 patients were compared with those from cell lines and with the median leucocyte mRNA expression profile of 53 HBDs [26, 27], to define tumor cell related gene expression in patient EVs. The mRNA expression profiles were generated for all cell lines except for MDA-MB-435s.

### Genomic DNA contamination, mRNA target gene transcript quantification and mutation detection by digital PCR

The variant allele frequencies (VAF) and number of mutant and wildtype (EV-) RNA transcripts were evaluated in both cell line and patient EV-RNA samples for 4 known oncogenes using the QuantStudio 3D digital PCR system (Thermo Fisher, Cat. No: A29738). The used mutation-specific TaqMan assays, summarized in Table S1, were: PIK3CA p.H1047R(c.3140A > G) and p.H1047L(c.3140A > T), KRAS Screening Kit, KRAS p.G12C(c.34G > T), p.G12D(c.35G > A) and p.G12V(c.35G > T), EGFR p.T790M(c.2369C > T). In addition, BRAF was evaluated by a multiplex of the assays BRAF wild-type and p.V600E(c.1799 T > A), and custom made exon spanning BRAF p.V600E(c.1799 T > A) assay next to BRAF p.V600E(c.1799_1800delinsAA) and p.V600K(c.1798_1799delGTinsAA). Taqman expression assays were used with a FAM labeled MGB-probe for *PIK3CA*, *KRAS*, *BRAF* or *EGFR* and multiplexed with the VIC labeled MGB-probe for *GAPDH* (Cat. No: 4326317E). All dPCR assays were performed on the ProFlex 2 x flat PCR System (Thermo Fisher, Cat. No: 4484078) thermal cycler in combination with the QuantStudio 3D Digital PCR Chip Adapter Kit (Thermo Fisher, Cat. No: 4485513) using the following program: 1 cycle of 10 min at 96 °C, 40 cycles extension/annealing of 2 min at 56 °C for the Thermo Fisher assays or at 52 °C for the Bio-Rad assays, 30s at 98 °C and 1 cycle of 2 min at 56 °C and terminated at 10 °C. After amplification, data were acquired using the Quantstudio 3D dPCR instrument and analyzed with the web-based Quantstudio 3D dPCR Analysis Software version 3.01 (Thermo Fisher). At least one positive and one no-template control (RNAse-free Water) were used for each assay to determine the thresholds for calling positive mutant and wildtype copies. The software automatically calculated the VAF by dividing the number of mutant copies by the total measured copies (wildtype + mutant). Presence of mutant EV-RNA transcripts was determined for the cell lines BT20, MDA-MB-231 and MDA-MB435s but not for MDA-MB-361 and MCF-7 for which the PIK3CA p.E545K mutation detection assay failed on EV-RNA.

### Statistical analysis

Protein levels of EpCAM, APOB, FLOT-1 and CD9 were evaluated in Patient and HBD derived EVs and tested for statistical differences using the two-tailed T-test. *P*-values lower than 0.05 were considered significant. To identify differentially expressed genes between RNA isolated from EVs and tumor cells, a paired class comparison analysis was performed on the generated 96 genes expression profiles of both cellular RNA and EV-RNA using BRB-ArrayTools version 4.5.0 (“http://linus.nci.nih.gov/BRB-ArrayTools.html“). False discovery rate (FDR) was set at 10% to correct for multiple testing and the significance threshold of the univariate tests was set to *P*-value < 0.05 ^33^. Genes that passed these criteria were considered differentially expressed.

## Results

Our study evaluated EV preparations of plasma, pre-analytical conditions on CD9-positive EVs amounts and the downstream analysis of EV-RNA in cell line models, healthy blood donors (HBDs) and two cohorts of patients, and describes a strategy to detect ctEVs in plasma by mutant transcripts and gene expression.

### Characterization of EV preparations

**Fig. 2:**
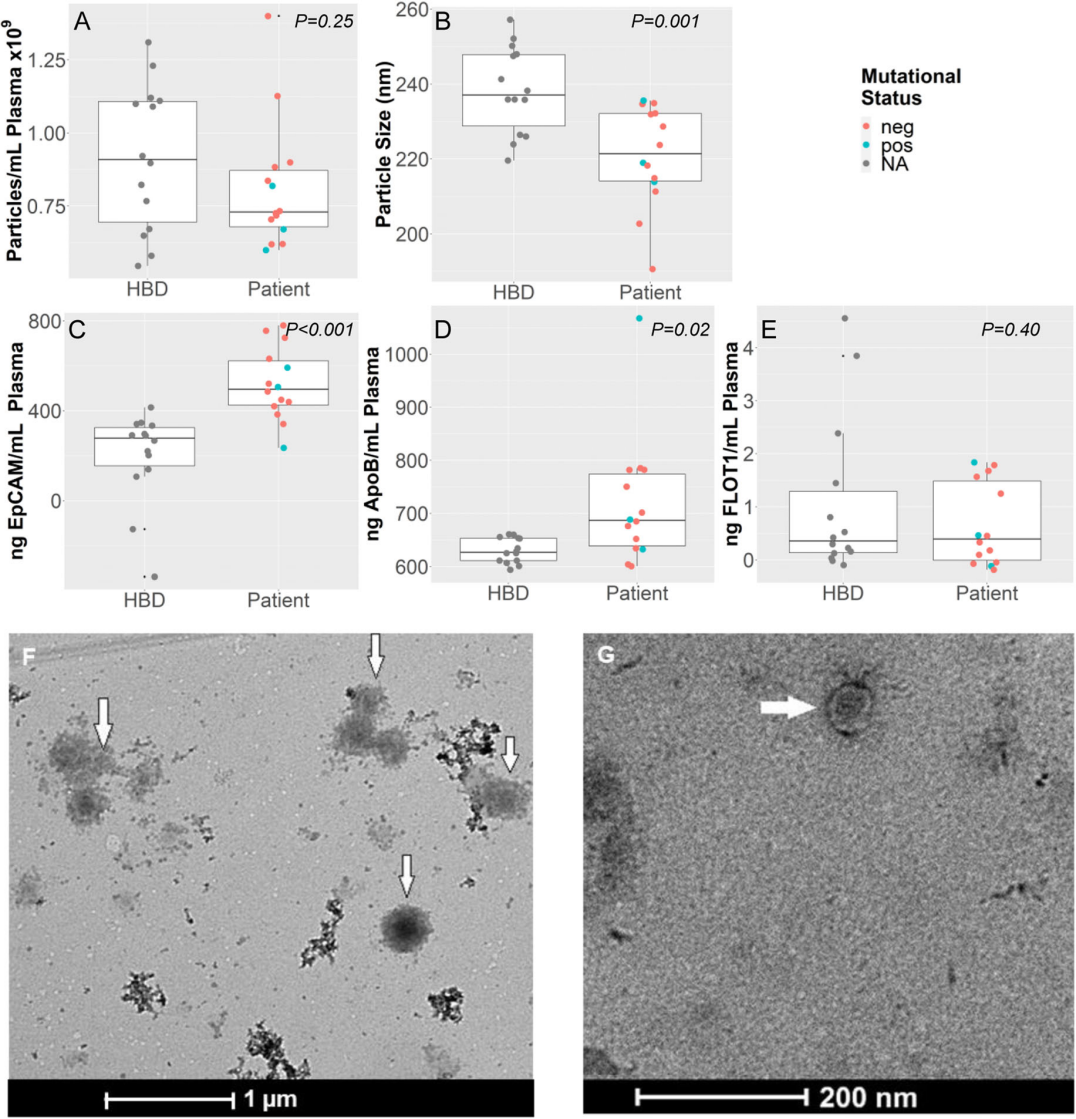
Characterization of EV-preparations. Plasma EV-preparations from patients and HBDs containing EVs isolated by the Exo (RN) easy kit (Qiagen) were characterized by NTA (**a**,**b**), immune-assays (**c**-**e**), and TEM (**f**, **g**)). Figures **a**-**b** demonstrate differences in nanoparticle concentration (**a**) and size (**b**) between healthy blood donors (HBD) and patients (PT). Figures C-E illustrate the EV protein content measured for EpCAM, APOB and FLOT-1. EV-preparation of patients have more EpCAM but less APOB compared to HBDs. The EV cytosolic protein FLOT-1 was comparable between both EV-preparations. Figures **f** and **g** shows EVs, indicated by arrows, as visualized by TEM from EV-preparations of patient 15 plasma. Pictures were taken at 11 k (**f**) and 45 k (**g**) magnification

The EVs of plasma from solid cancer patients and HBDs were harvested by the ExoEasy protocol and were evaluated for specific characteristics described by MISEV2018 guidelines (Fig. 2). No significant differences in EV preparations between patients and HBDs were observed for nanoparticle concentration (*P* = 0.25) and EV protein analysis of FLOT1(*P* = 0.31) but a significant difference was observed for nanoparticle size(*P* = 0.001), and for protein levels of APOB(*P* < 0.02) and of EpCAM (*P* < 0.001) (Fig. 2a-e). TEM demonstrated the presence of EVs as exemplified for a patient preparation (Fig. 2f, g).

### Pre-analytical conditions and amounts of CD9-positive EVs

**Fig. 3:**
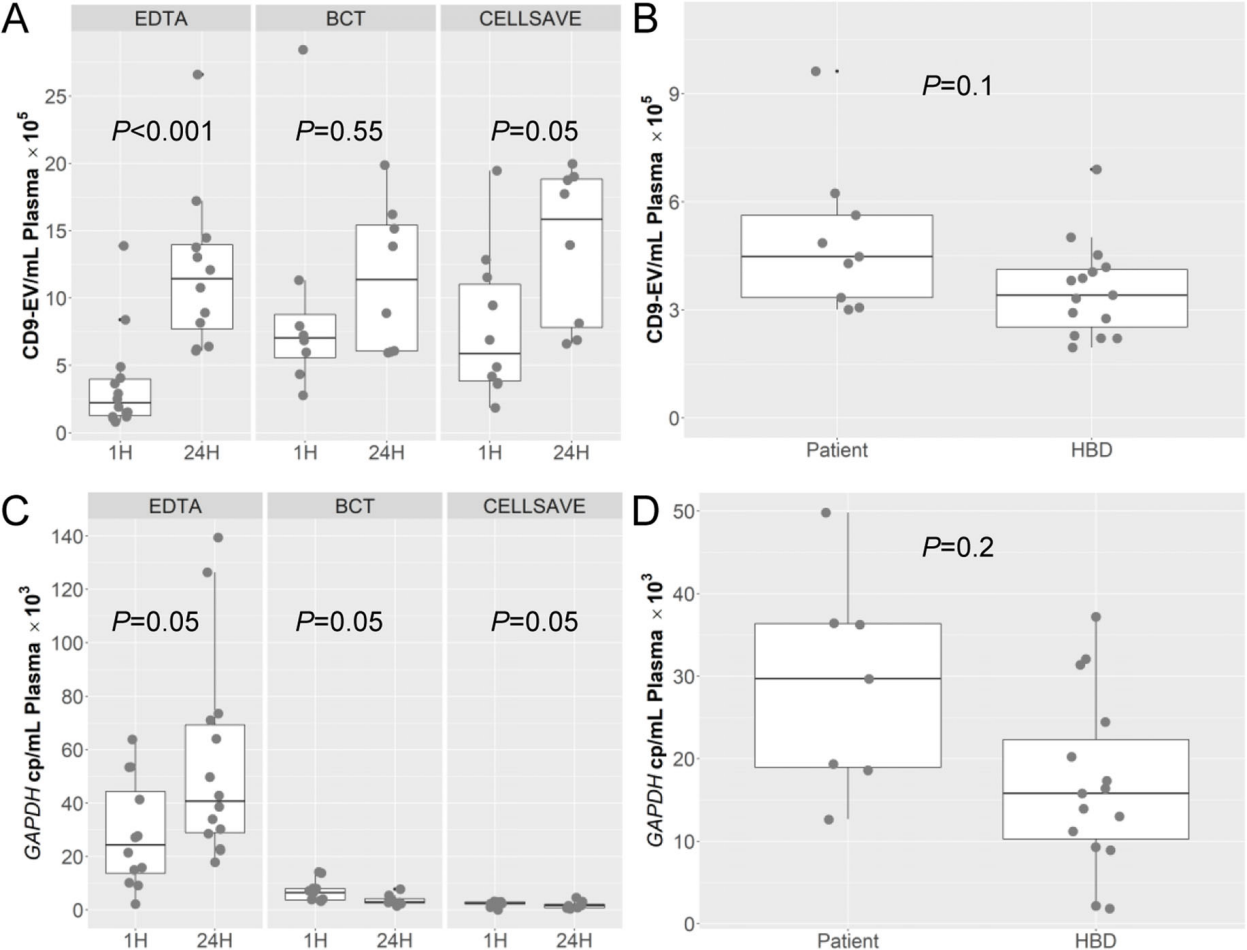
Analysis of CD9 and *GAPDH* expression in EVs from plasma of cancer patients. CD9-EV levels on EVs were measured by TRiFIC and *GAPDH* transcripts in EV-RNA were determined by digital PCR. The boxplots shows for 8 patients of cohort 1 in **a**) CD9 levels per mL plasma and **c**) *GAPDH* copies/mL plasma measured in plasma collected in different tubes (EDTA, BCT and CellSave) and processed at two time points (1 h and 24 h). For the second cohort of 8 patients and 15 Healthy Blood Donor (HBDs) are boxplots presented in **b**) CD9 levels per mL plasma and **d**) *GAPDH* copies/mL plasma both collected in EDTA tubes and processed within 24 h (< 24 h) from the blood draw. CD9 measurements were performed in duplicate

Extracellular vesicles derived from conditioned cell culture medium and from plasma of 15 HBDs and 8 patients were quantified by the TRIFic CD9 assay. The CD9 levels in conditioned medium from all cell lines were well above (≥1.4×) the levels measured in culture medium alone (Fig. S1). Fetal Bovine Serum (FBS) gave no signal, confirming the CD9 antibody specificity for human EVs (data not shown). The plasma CD9 levels in cohort I of 8 patients were compared between blood collected in EDTA, BCT or CellSave tubes and processed into plasma at 1 h or at 24 h after blood draw. For samples processed at 1 h, CD9 levels were significantly higher for BCT and CellSave tubes (both *P* < 0.008) compared to standard EDTA tubes (Fig. 3a). All tube types had higher CD9 levels in plasma processed at 24 h compared to plasma processed at 1 h (*P* < 0.05) (Fig. 3a). Higher CD9 levels were also observed in patients of cohort II compared to HBDs in blood of EDTA tubes processed into plasma within 24 h (< 24 h), however, not significantly different (Fig. 3b). Finally, CD9 levels in EDTA tubes gradually increased with time to process blood into plasma after venipuncture (*P* < 0.05) (Fig. S2A).

### Comparison of EV-RNA and matched tumor cell mRNA gene expression profiles

**Fig. 4:**
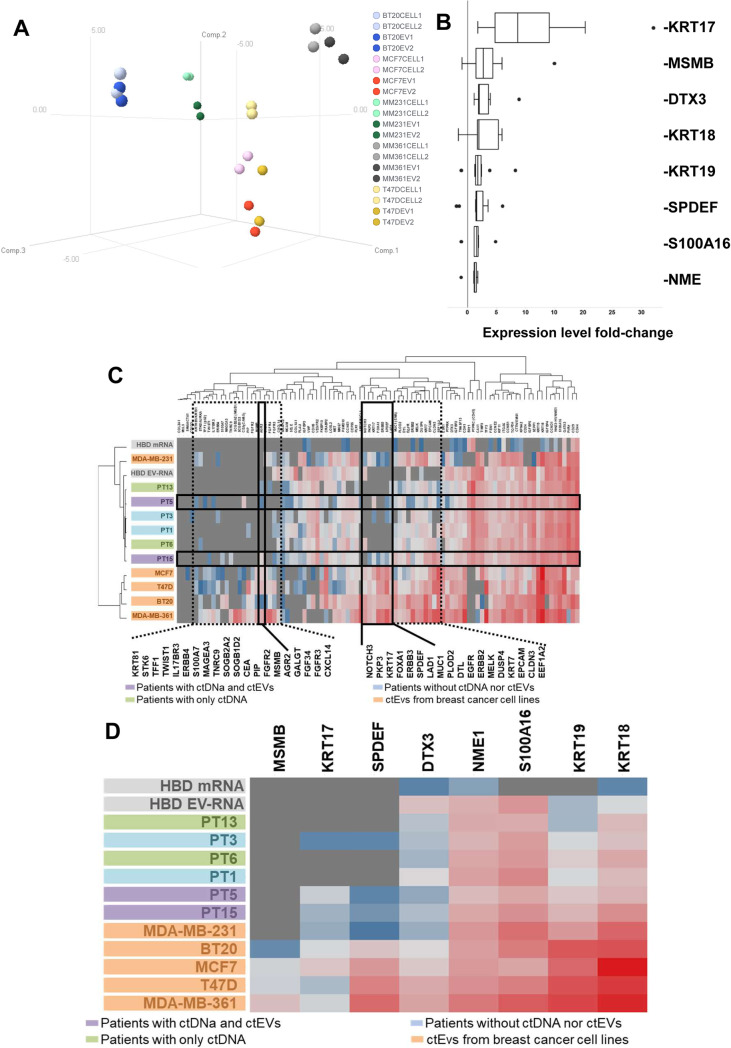
Gene expression profiling of EV-RNA. Expression of 96 genes was evaluated by RT-qPCR in EV-RNA and cellular mRNA from cancer cell lines and HBDs and EV-RNA from patients. **a** 3D Principal Component analysis plot of the 96 gene expression profiles in EVs and matching tumor cells from 5 breast cancer cell lines. Each dot represents a sample. Axes show the first three principal components. Dark colored dots indicate the EV-RNA samples and the lighter colors the cellular mRNA. **b** Enriched genes in EVs are indicated on the Y-axis while the X-axis shows the expression fold-change in EV-RNAs compared to their matched cellular mRNA from 5 breast cancer cell lines. Each boxplot consists of data from duplicate analysis of EV-RNA and tumor cell mRNA. **c** Hierarchical cluster analyses of EV-RNA expression profiles from patients, HBD and breast cancer cell lines compared to median HBD leucocyte mRNA expression. Clustering is shown for 96 genes (upper plot) and for the 8 genes enriched in tumor cell line EVs (lower plot; see also Fig. 2b). The boxes in the upper plot indicate genes upregulated in EVs from tumor cells compared to HBDs (dashed black boxes), and tumor cell related genes (solid black boxes) expressed in cell lines and in patients with ctEVs and ctDNA (PT5 and 15). Expression levels of target genes were compared to the average of reference genes *HMBS*, *HPRT1* and *GUSB* levels; grey color indicates no expression, blue color is below reference gene level, and red color is above reference gene level

To investigate whether the transcriptome was equivalent between EVs retrieved from conditioned medium and the cells they originated from, we compared the expression of 96 genes in 5 breast cancer cell lines and their respective EVs. The authentication of cell lines was established by STR analysis (Fig. S3A) and included both basal (MDA-MB-231, BT20) and luminal (MCF7, T47D, MDA-MB-361) molecular breast cancer subtypes. The 96 gene expression profiles of EV-RNA and matched parental cell line mRNA correlated highly (*R*_*Pearson*_ > 0.85, *P* < 0.05; Table S2), and grouped together after hierarchical clustering (Fig. S3B) and principal component analysis (PCA) for all cell lines except T47D (Fig. 4a). Although expression profiles between cell lines and matched EVs were highly comparable, paired class comparison analyses revealed that the expression levels of 38 genes (Fig. S4 and Table S3) were different between EVs and tumor cells (*P* < 0.05). Specifically, 8 genes (*DTX3, KRT17 KRT18, KRT19, MSMB, NME1, S100A16, SPDEF*) were enriched in EVs (Fig. 4b).

### Workflow for gene expression and mutant transcript analysis in EV-RNA

**Fig. 5:**
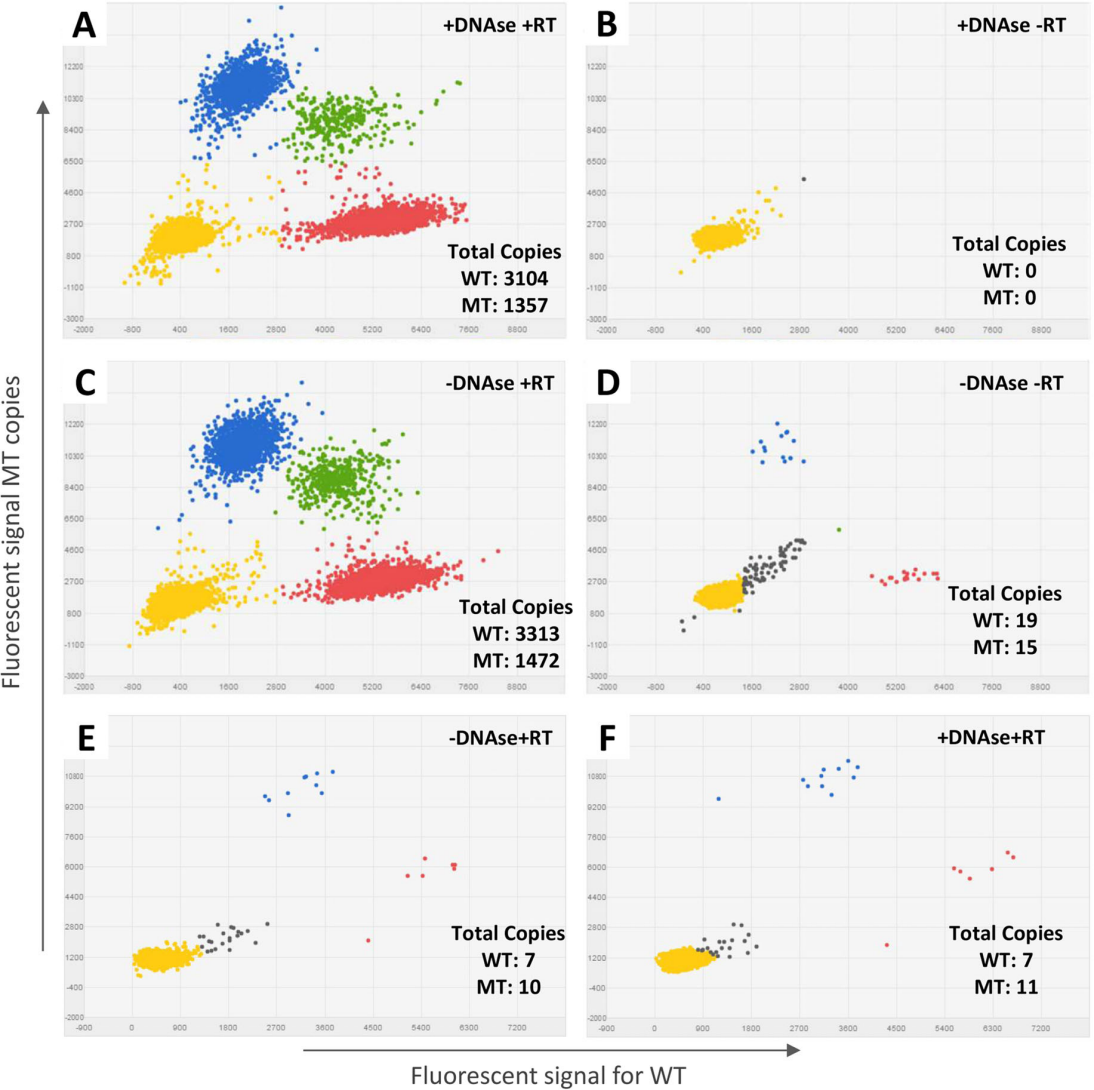
Mutation analysis by dPCR in (EV) RNA with and without DNAse treatment. Dot-plots in panel **a**-**d** indicate the presence of wild-type (WT) and/or mutant (MT) *PIK3CA* p.H1047R copies in BT20 mRNA treated **a**) with DNase and reverse transcriptase (RT), **b**) with DNAse and without RT, **c**) without DNase and with RT, **d**) without both DNAse and RT. Dot-plot in panel **e**-**f** indicate the presence of wild-type (WT) and/or mutant (MT) of *EGFR* p.T790M in patient 15 EV-RNA **e**) treated without DNAse and **f**) with DNAse treatment. Similar mutant and wild-type copies were observed in both conditions. Blue: wells with mutant copies, Red: wells with wild-type copies, Green: wells containing both wild-type and mutant copies, Yellow: empty wells, Grey: undetermined wells

Next, we tested our workflow for mutant and wildtype transcript detection in cellular mRNA and EV-RNA using the QuantStudio 3D digital PCR and Taqman mutation assays for *KRAS* p.G12D and p.G13D, *PIK3CA* p.H1047R, *EGFR* p.T790M and *BRAF* p.V600E. We designed an exon spanning *BRAF* p.V600E assay for RNA templates only. All other mutation assays amplified both DNA and RNA; for a proper evaluation of RNA templates only with these assays a DNase treatment prior cDNA synthesis was performed to remove any DNA templates. As proof-of-principle, we demonstrated that DNAse treatment successfully removed all remaining DNA content by using the *PIK3CA* p.H1047R mutation assay on BT20 cell line mRNA (Fig. 5). Mutant and wild-type copies were detected in cellular mRNA specimens treated with/without DNase (+DNAse/−DNAse) after reverse transcription (+RT) (Fig. 5a and c) whereas no *PIK3CA* copies were generated in the sample with DNase and without RT enzyme (Fig. 5b). Furthermore, few mutant and wild-type copies were measured when the sample was not converted into cDNA (minus RT) without DNase treatment (Fig. 5d) indicative of limited contamination for the specimen with cellular germ-line DNA. Similarly, in EV-RNA of patient 15 which harbors a *EGFR* p.T790M mutation no difference was found in the number of mutant and wild-type copies between samples with and without DNase (Fig. 5e and f). Finally, analysis of a genomic fragment within the intronic region of *TK1* confirmed the successful removal of any residual DNA after DNase treatment in both cellular mRNA and patient EV-RNA (Fig. S5).

**Fig. 6:**
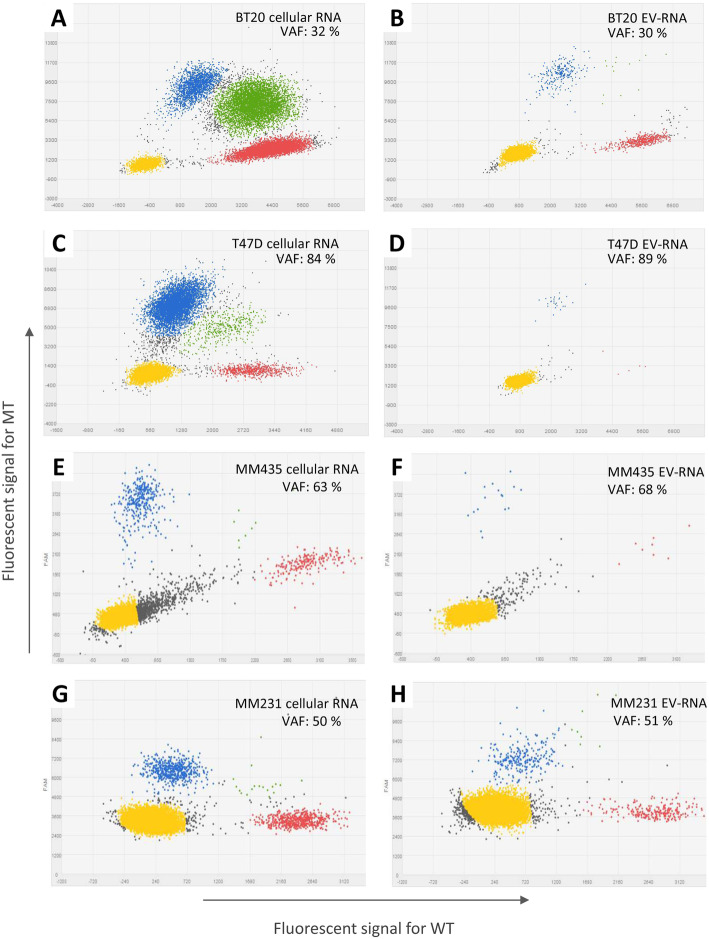
Mutation analysis of human breast cancer cell lines by digital PCR. The figure shows dPCR dot plots for cellular mRNA (**a**, **c**, **e** and **g**) and matched EV-RNA (**b**, **d**, **f** and **h**) indicating wild-type (WT) and mutant (MT) copies for breast cancer cell lines BT20 (*PIK3CA* p.H1047R), T47D (*PIK3CA* p.H1047R), MDA-MB-435 (*BRAF* p.V600E^(c.1799T > A)^) and MDA-MB-231 (*KRAS* p.G13D). Dots represent wells with mutant copies (blue), wild-type copies (red), both wild-type and mutant copies (green), empty wells (yellow), and undetermined wells (grey). VAF: Variant Allele Frequency

Next, the *PIK3CA* p.H1047R mutation status analyzed by dPCR was compared between cellular mRNA and matched EV-RNA of BT20 and T47D cell lines. Mutant transcripts were present at comparable frequencies between EV-RNA and matched cellular mRNA (Fig. 6; BT20: 30% vs 32% and T47D: 89% vs 84%, respectively). Similar results were obtained in in MBA-MB-435 for the *BRAF* p.V600E in cellular RNA and EV-RNA (68% vs 63%, respectively) and in MDA-MB-231 for the *KRAS* p.G13D mutation detected in cellular RNA and matched EV-RNA (51% vs 50%, respectively).

### Analysis of gene expression and mutant transcripts in patient EV-RNA

The feasibility of above workflow to quantify gene expression together with wildtype and mutant transcript levels was also evaluated in EVs derived from minute amounts of plasma (< 1 mL) from patients with various solid cancers. In all EDTA samples at 1 h and 24 h of the cohort I patients, expression of the reference gene *GAPDH* was detectable in EV-RNA with a median of 34.5 × 10^3^ and 53.4 × 10^3^ copies/mL plasma_,_ respectively (Fig. 3c). Up to ten-fold lower *GAPDH* levels (average median 3.06 × 10^3^ copies/mL plasma) were measured in both BCT and CellSave tubes independent of their processing time-point (Fig. 3c). In addition, no significant increase was observed in *GAPDH* copies/mL plasma in EDTA-blood samples processed within 24 h (Fig. S2B). For cohort II, EDTA blood from patients collected within 24 h had a median of ~ 24.4 × 10^3^ of *GAPDH* copies/mL plasma, which was 1.55-fold higher than the levels measured in plasma derived from HBDs (Fig. 3d).

Next, the number of transcripts was established by dPCR and gene expression assay for the target gene in which a mutation was reported (Table 1 & Table S1). Target gene copies were only detected in EDTA tubes (Table 1) but not in BCT nor in CellSave tubes (data not shown). For this reason, only results obtained in EDTA tubes of cohort I and II were reported.

**Fig. 7:**
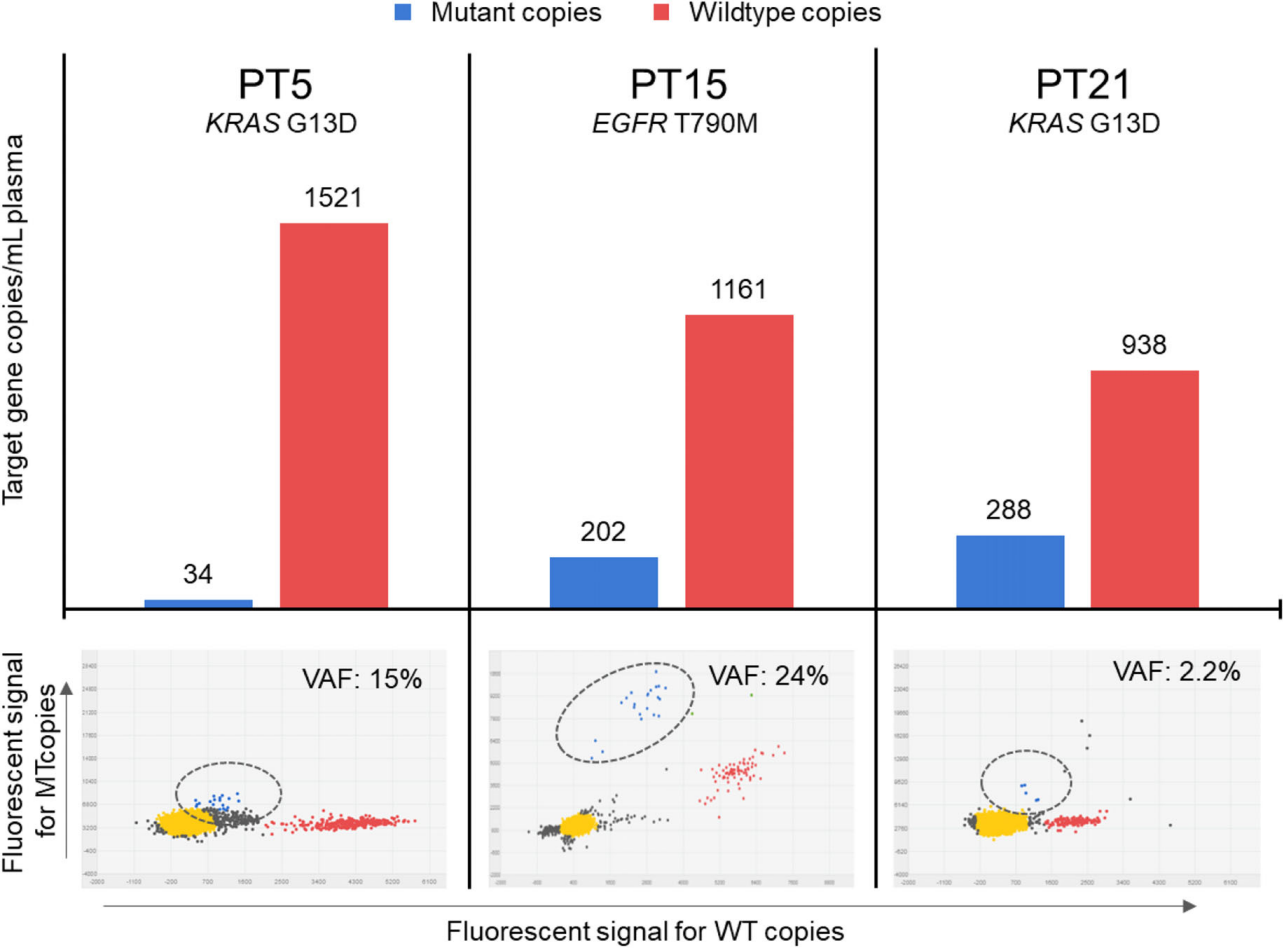
Patients in which mutant target gene copies were detected in EV-RNA. The top part of the figure show blue bars which indicate the number of mutant copies/mL plasma and red bars that indicate the number of wild-type copies/mL plasma. Additionally, the bottom section of the figure show the results of the dPCR including the variant allele frequencies. Blue: represent mutant copies, Red: represent wild-type copies, Yellow: represent empty wells, Grey: represent undetermined wells, VAF: Variant Allele Frequency

Gene transcripts in EV-RNA were quantified with dPCR using both mutation and expression assay for the target gene with a somatic variant (Table 1 and Figs. S6 & S7). Both types of assays detected at least 100 copies per mL plasma of target gene EV-RNA transcripts in all cases, except for patients 2 and 13 with no or very few copies observed. Mutant target gene transcripts ranging from 34 to 288 copies per mL plasma, on the other hand, were only found in EV-RNA from patients 5 (*KRAS* p.G13D),15 (*EGFR* p.T790M) and 21 (*KRAS* p.G13D) with VAFs of 15, 24 and 2.2%, respectively (Table 1, Fig. 7).

The 96 gene expression profiling in EV-RNA was retrospectively performed in EV-RNA of 6 patients (2 NSCLC, 2 CRC, 1 BC, 1 cholangiocarcinoma) with enough material for the analysis and compared with those of the cancer cell lines and of leucocytes from HBDs. Hierarchical cluster analyses of these expression profiles for all genes but also for above 8 genes enriched in EVs, showed that EV-RNAs from patients clustered more closely to the HBDs than to the cancer cell lines. Only a few genes were specifically expressed in patients with (ct) EVs and in EVs of cancer cell lines. These genes included *AGR2, KRT17, SPDEF, and LAD1* which were expressed in EV-RNA of patients 5 and 15 and of all cancer cell lines, but not in HBDs (Fig. 4c).

## Discussion

Even though cfDNA has recently shown great promise, cfDNA analyses does not provide information on gene and protein expression which EVs likely can. For this purpose, we developed a workflow to isolate and characterize ctEVs derived from small amounts of plasma (< 1 mL) from 20 metastatic cancer patients with different types of solid tumors.

First, EV preparations of plasma from patients and HBDs were characterized to provide evidence that EVs were harvested with our workflow. It has already been reported that EVs were harvested from plasma of ovarian and prostate cancer patients using the membrane-based affinity binding step of the Exo (RNA) Easy kit and that NTA, TEM and immune assays can be used to characterize these EVs [27, 28].

To have an additional quantitative measure of the EVs in the analyzed specimens, we used the CD9 TRIFic assay to measure the CD9 transmembrane protein, which is enriched up to 10 times in EVs compared to other particles [24, 29–32]. The advantage of the CD9 TRIFic assay is that it can be easily used without plasma purification and is not hampered by other non-EV particles such as lipid particles and protein complexes. Furthermore, it is not restricted by vesicle size compared to other methods for EV quantification such as flow cytometry, flow immuno-detection (LFIA), nanoparticle tracking and tunable resistive pulse sensing [24, 33, 34]. The measured CD9 levels varied considerably between conditioned cell line media (Fig. S1), cancer patients and healthy donors but confirmed observations reported previously [24]. We also showed that processing time after blood draw affects CD9 levels in plasma samples (Fig. S2A). Noteworthy, CD9 levels were higher in all plasma samples processed at a later time point, suggesting that EVs are released by (blood) cells [35] during storage after venipuncture but before blood was processed into plasma. Finally, higher CD9 levels were measured (although not significant) in plasma of patients with cancer compared to HBDs, which was also previously described for prostate cancer patients [36]. Our study now demonstrated similar FLOT1 levels in EV preparations from patients and HBDs. The low levels of FLOT1 may suggest that FLOT1 is not highly expressed on the surface of a vesicle but rather on the inside as FLOT1 is found on the inner membrane of a cell. Therefore, we might speculate that during the formation of the EV’s by budding of the cell membrane FLOT1 remains inside an EV. Most studies involving measurement of FLOT1 performed this by means of western blotting, which involves a form of EV lysis making it possible to obtain higher amounts of FLOT1 [37–40]. Further studies are necessary to confirm these findings. Furthermore, higher APOB levels were observed between patient and HBD EV preparations and are only slightly lower compared to APOB levels directly measured in plasma (data not shown) indicating that the used method for EV purification still co-isolated a high amount of lipoproteins. This might hamper the recovery of EVs from plasma and negatively influence detection of mutations in EV-RNA. Nonetheless, EpCAM protein levels were significantly higher in patients’ EVs advocating for enrichment of ctEV using EpCAM as a target for capture. This might improve the purity of the EV preparations and also our ability to detect mutations due to a higher rate of ctEV recovery. However, further studies are needed to verify our findings and EpCAM based capturing methodologies should be explored.

Several studies reported that EVs are involved in tumorigenesis, proliferation, drug resistance, angiogenesis and the development of pre-metastatic nichesniches [19, 41–44]. The observation of EV-derived RNA being translated into functional proteins in recipient cells shows that (tumor) cells use EVs to deliver information to other cells [45–47]. Our gene expression profiling of cellular mRNA and EV-RNA demonstrated that EVs carry multiple gene transcripts and cluster with their matched cellular counterpart. This suggests that EV-RNA express molecular (sub) type specific genes as previously shown by others thers [48, 49]. Interestingly, 8 genes were more abundant in EV-RNA compared to cellular RNA which might be an indication of selective enrichment (e.g. several KRTs) and/or exclusion of certain transcripts from EVs. Two of these EV-RNA abundant genes (*KRT17, SPDEF*) were also expressed in patients 5 and 15 with ctEVs whereas not expressed in healthy blood donor EV-RNA and mRNA. The gene *SPDEF* was previously reported as part of a urine exosome gene expression assay which also includes the genes ERG and PCA3. This 3-gene assay was reported to discriminate high-grade prostate cancer from low-grade and benign diseaseisease [50]. Likewise, KRT17 and SPDEF might be used to discriminate between patients with high and low tumor-load as indicated by the measured primary tumor percentage and cfDNA VAF (Table 1). Additionally, our findings suggest that such an EV gene expression assay might also be applicable in plasma EV from patients with other malignancies. However, we did not find a real difference between EVs from patients and those derived from healthy donors while more differences were observed with EVs from tumor cell lines. We might speculate that EVs are mostly derived from healthy tissue as previously described by Mitchell et al. These authors showed no real difference in EVs quantity in urinary-exosome between healthy men and those with locally advanced diseadisease [51]. However, further studies are needed to validate our findings as well as to develop methods to enrich for tumor-derived EVs.

The use of EVs as liquid biopsy biomarker for cancer patients is challenged by pre-analytical factors, like time to process blood into plasma and type of blood collection tubes usedused [7, 52–54]. Several studies have shown the impact of anticoagulants (such as sodium-citrate, EDTA or heparin) on EV analysis outcomutcome [7, 54]. Our aim was the simultaneous analysis of both cfDNA and EV-RNA. Therefore, we investigated EDTA, BCT, and CellSave tubes with the latter two tubes showing stable cfDNA quality and quantity in blood processed into plasma up to 24 h after venipuncture [23]. However, the results of our workflow show that BCT and CellSave tubes are less suited for analyses of EV-RNAs. Recently, BCT-RNA tubes were reported to stabilize cell-free RNA (cfRNAcfRNA) [55], which might also be feasible for EV-RNA but due to our retrospective study, was not evaluated.

Next, we showed in EDTA tubes the impact of time delay between blood sampling and plasma processing on EVs and EV-RNA. Ideally, blood should be processed into plasma immediately after blood draw, which is not feasible in daily clinical practice and in multicenter clinical trials. We observed increased EV-RNA *GAPDH* transcripts with time to process blood into plasma after blood draw. This increase was previously also reported for cfRNA copies of *GAPDH* and beta-2-microglobulin (*B2M*) [55], showing that processing time affects downstream EV-RNA. However, the observed increase of *GAPDH* transcripts within 24 h was moderate and not significantly different compared to the number of *GAPDH* transcripts after 1 h processing. The 24 h processing of blood is more feasible with current clinical practice.

Previous studies detected tumor-specific mutations and translocations in DNA and RNA from EVs, respectivetively [56–59]. We now evaluated EV-RNA of 20 cancer patients for somatic variants found in tissue and in 17 cases of these also in plasma cfDNA. The sensitivity for mutation detection in plasma EV-RNA (3/20 patients; 15%) was considerably lower than for cfDNA (17/20 patients; 75%). This discrepancy in mutation detection between cfDNA and EV-RNA can be partially due to differences in time of plasma storage and analyses, plasma input used (i.e. less than 1 mL was available and evaluated for EV-RNA whereas 3 mL plasma was used for cfDNA analyses [23]), on the presence target gene copies and whether a mutation is expressed or not. Mutation detection in EV-RNA might improve with the enrichment of cancer- and/or tissue-specific EVs, when more plasma is available for such analyses. Moreover, not all hotspot mutations in DNA result in mutant transcripts carried by EVs, which might also explain the discrepancy in detection frequency between ctDNA and ctEV. In this context, we have used a very strict definition for ctEV by indicating EVs that carry mutations in their EV-RNA. In fact, although our EpCAM ELISA analyses showed many more cases with EpCAM-positive EVs, which could have been defined as ctEV, most of them did not carry detectable mutant transcripts. Interestingly, mutant transcripts were detected in all patients with high amounts of ctDNA (> 20% ctDNA; patients 5, 15 and 21) except one (patient 8). This suggests that also the tumor load in plasma might be important for successful mutant transcript detection. Further studies are needed to determine whether mutant transcripts are detectable in patients with low amounts of ctDNA, without or after enrichment of cancer -specific EVs.

Nowadays, several studies have highlighted the clinical value of EVs in providing information for real-time monitoring of disease due to their minimal invasiveness as well as the opportunity to characterize the status of the tumor by using their content, which includes proteins, DNA and RNA. In this context, mutated mRNAs in plasma EVs are currently being used for the assessment of both hematological as well as solid tumors, such as prostate, lung and other solid tumors [60]. For example, mutation in the tumor-specific mRNA of epidermal growth factor receptor was detected in EVs from serum of patients with glioblastoma [14]. EV-RNAs are therefore a snapshot of the content and state of the cells that secrete them. Compared to circulating RNA (cfRNA), RNAs cargo in extracellular vesicles are quite safe from degradation by RNases enzymes even if it is still unclear what percentage of RNA cargo in EVs is functional or nor when transferred in the recipient cell [60].

Moreover, the antigenic markers on the EVs surface make it possible to discriminate the cells from which they were derived, allowing enrichment of vesicles from a particular tissue source, such as a tumor tissue. Many new sensitive technologies, such as digital PCR (dPCR) or NGS are being used to enhance detection of specific RNA species in extracellular vesiclesicles [61]. However, the fact that with our work we cannot really discriminate the subset of extracellular vesicles derived from a cancer tissue from other vesicles is a limit for their clinical application. Similarly, an issue concerns the choice of the best method to isolate and characterize cancer EVs from all the other vesicles which are released from the (normal) cell sources [62]. Nonetheless, our study shows a relatively fast method for obtaining EVs, generating expression profile and perform mutation analysis (with the known present limitations). However, to overcome the present limitations and enhance the clinical application of EVs for tumor management, further investigations are still needed.

## Conclusions

We provide a workflow for the detection of ctEVs by a spin column-based generic isolation of EVs. The workflow was followed by PCR-based measurements of gene expression and mutant transcripts in EV-RNA derived from cancer patients’ blood plasma processed within 24 h. Tumor-specific mutations in blood, however, were less often observed in EV-RNA than in cfDNA.

## Supplementary Information

**Additional file 1: Fig. S1.** Analyses of CD9-EV levels by TRIFic™ exosome assay. **Fig. S2.** TRiFIC™ Analysis of CD9-EV in plasma of cancer patients and *GAPDH* copies in their EV-RNA. The boxplots shows in A) CD9-EV levels per mL plasma and in B) the *GAPDH* copies/mL plasma measured in EDTA tubes and processed at three time points (1 h, within 24 h and 24 h) for 8 patients from each cohort 1 (at 1 h and 24 h) and cohort 2 (< 24 h). All measurements for CD9-EV were performed in duplicate. **Fig. S3.**A: Cell line STR Authentication measured by Powerplex 16 (Promega, cat: DC6531) of cell lines obtained from ATCC. **Fig. S3**B - Expression profiling of breast cancer cell line mRNA and EV-RNA. TaqMan Gene Expression Assays for 96 genes were used to evaluate the RNA expression levels by real time RT-PCR. The measurements were performed in duplicate on RNA from both cell line and EVs. Complete linkage cluster analysis was performed for both cell line mRNA and matched EV-RNA and demonstrated that EV expression profiles are similar to their parental cell line profiles. Each horizontal row represents a gene, and each vertical column corresponds to a sample with numbers indicating a separate analysis (1 and 2). Arrows at the right of the figure indicate the genes which are upregulated in EVs. Expression levels are colored at median (white), above median (red) or below median (blue). **Fig. S4.** Differentially expressed genes in EV-RNA compared to their matched cellular mRNA. The data are based on the independent duplicate analysis of 96 genes, but the figure shows only the 38 genes which overall were significantly different between EV-RNA and cellular RNA. Genes are indicated in the first column. The green bars for the individual cell lines represent expression levels of each gene relative to 3 reference genes (*HMBS*, *HPRT1* and *GUSB*) for two independent measurements of EV-RNA (EV1, EV2) and of cellular RNA (CR1, CR2). The difference in expression levels between EV-RNA and cellular RNA are shown by bars in red (EV > CR) or in blue (EV < CR). The first eight genes are more abundant in EV-RNA than in cellular RNA for almost all five cell lines. **Fig. S5** - Analysis of an intronic region of *TK1* on cellular EV-RNA and patient EV-RNA. The presence of DNA was evaluated in A) Celline DNA (positive control) and cellular EV-RNA treated in B) with DNAse and Reverse Transcriptase (RT); in C) with DNAse and without RT; in D) without DNAse and with RT; in E) without both DNAse and RT; and in patient EV-RNA treated in F) with DNAse and RT; in G) without DNAse and with RT; and H) show results for the no template control. No DNA was detected in both cellular and patient EV-RNA treated with DNAse. Blue: represent *TK1* copies, Yellow: represent empty wells, Grey: represent undetermined wells, VAF: Variant Allele Frequency. **Fig. S6**. Digital PCR dot-plots of target genes in 20 patients. Digital PCR dotplots of target genes in 20 patients. The dPCR-plots for each of mutation assays are presented for cohort 1 of EDTA plasma at 1 h (12 patients: # 1–16) and for cohort 2 of EDTA plasma within 24 h (8 patients: #17–24). Dots represent wells with mutant copies (blue), wild-type copies (red), both wild-type and mutant copies (green), empty wells (yellow), and undetermined wells (grey). PT: Patient; POS ctrl: Positive Control. **Fig. S7**. Mutation analyses of target genes in EV-RNA of 12 patients derived from EDTA 24 h plasma. Wild-type and mutant copies/mL plasma measured by dPCR from EDTA plasma processed at 24 h after blood draw from the cohort 1 cancer patients.

**Additional file 2: Table S1.** Assays used for expression analyses and for wild-type and mutant copies of target genes.

**Additional file 3: Table S2.** Pearson correlation coefficient R based on expression levels of a 96 gene-panel.

**Additional file 4: Table S3.** Differentially expressed genes in EV-RNA compared to cell line mRNA.

## Data Availability

The datasets used and/or analysed during the current study are available from the corresponding author on reasonable request.

## Abbreviations

cfDNA: Cell-free DNA
ctDNA: Circulating tumor DNA
ctEVs: circulating tumor Extracellular Vesicles
dPCR: Digital polymerase chain reaction
EVs: Extracellular Vesicles
EV-RNA: Extracellular Vesicle RNA
HBDs: Healthy Blood Donor
NTA: Nanoparticle Tracking Analyses
RT: Reverse Transcriptase
TEM: Transmission Electron Microscopy
VAF: Variant allele frequency

## Human genes

BRAF: B-Raf proto-oncogene, serine/threonine kinase
EGFR: Epidermal growth factor receptor
KRAS: Kirsten rat sarcoma viral oncogene homolog
PIK3CA: Phosphatidylinositol-4,5-bisphosphate 3-kinase catalytic subunit alpha
GAPDH: Glyceraldehyde 3-phosphate dehydrogenase
TK1: Thymidine kinase 1
