# Supplementary material for: Detection of tumor-derived extracellular vesicles in plasma from patients with solid cancer
Source: BMC Cancer. 2021 Mar 24;21:315. doi: 10.1186/s12885-021-08007-z (PMC7992353; doi:10.1186/s12885-021-08007-z)
Supplement: Supplementary file 1 — Additional file 1: Fig. S1. Analyses of CD9-EV levels by TRIFic™ exosome assay. Fig. S2. TRiFIC™ Analysis of CD9-EV in plasma of cancer patients and GAPDH copies in their EV-RNA. The boxplots shows in A) CD9-EV levels per mL plasma and in B) the GAPDH copies/mL plasma measured in EDTA tubes and processed at three time points (1 h, within 24 h and 24 h) for 8 patients from each cohort 1 (at 1 h and 24 h) and cohort 2 (< 24 h). All measurements for CD9-EV were performed in duplicate. Fig. S3.A: Cell line STR Authentication measured by Powerplex 16 (Promega, cat: DC6531) of cell lines obtained from ATCC. Fig. S3B - Expression profiling of breast cancer cell line mRNA and EV-RNA. TaqMan Gene Expression Assays for 96 genes were used to evaluate the RNA expression levels by real time RT-PCR. The measurements were performed in duplicate on RNA from both cell line and EVs. Complete linkage cluster analysis was performed for both cell line mRNA and matched EV-RNA and demonstrated that EV expression profiles are similar to their parental cell line profiles. Each horizontal row represents a gene, and each vertical column corresponds to a sample with numbers indicating a separate analysis (1 and 2). Arrows at the right of the figure indicate the genes which are upregulated in EVs. Expression levels are colored at median (white), above median (red) or below median (blue). Fig. S4. Differentially expressed genes in EV-RNA compared to their matched cellular mRNA. The data are based on the independent duplicate analysis of 96 genes, but the figure shows only the 38 genes which overall were significantly different between EV-RNA and cellular RNA. Genes are indicated in the first column. The green bars for the individual cell lines represent expression levels of each gene relative to 3 reference genes (HMBS, HPRT1 and GUSB) for two independent measurements of EV-RNA (EV1, EV2) and of cellular RNA (CR1, CR2). The difference in expression levels between EV-RNA and cellular RNA are show [file 12885_2021_8007_MOESM1_ESM.docx]

**Detection of tumor-derived extracellular vesicles in plasma from patients with solid cancer**

**Authors:**, Silvia R. Vitale^1,4^*, Jean A. Helmijr^1^*, Marjolein Gerritsen^1^, Hicret Coban^1^, Lisanne F. van Dessel^1^, Nick Beije^1^, Michelle van der Vlugt-Daane^1^, Paolo Vigneri^4^, Anieta M. Sieuwerts^1,†^, Natasja Dits^2^, Martin van Royen^2^, Guido Jenster^2^, Stefan Sleijfer^1,3^, Martijn Lolkema^1^, John W.M. Martens^1,3^, Maurice P.H.M. Jansen^1^*

**Supplementary figures**


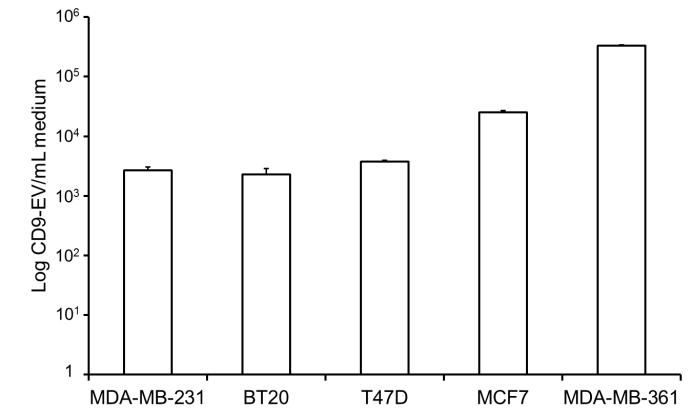


**Figure S1 - Analyses of CD9-EV levels by TRIFic™ exosome assay.**

The bar chart shows the log of CD9-EV counts per mL cell culture medium measured in five breast cancer cell lines.

**
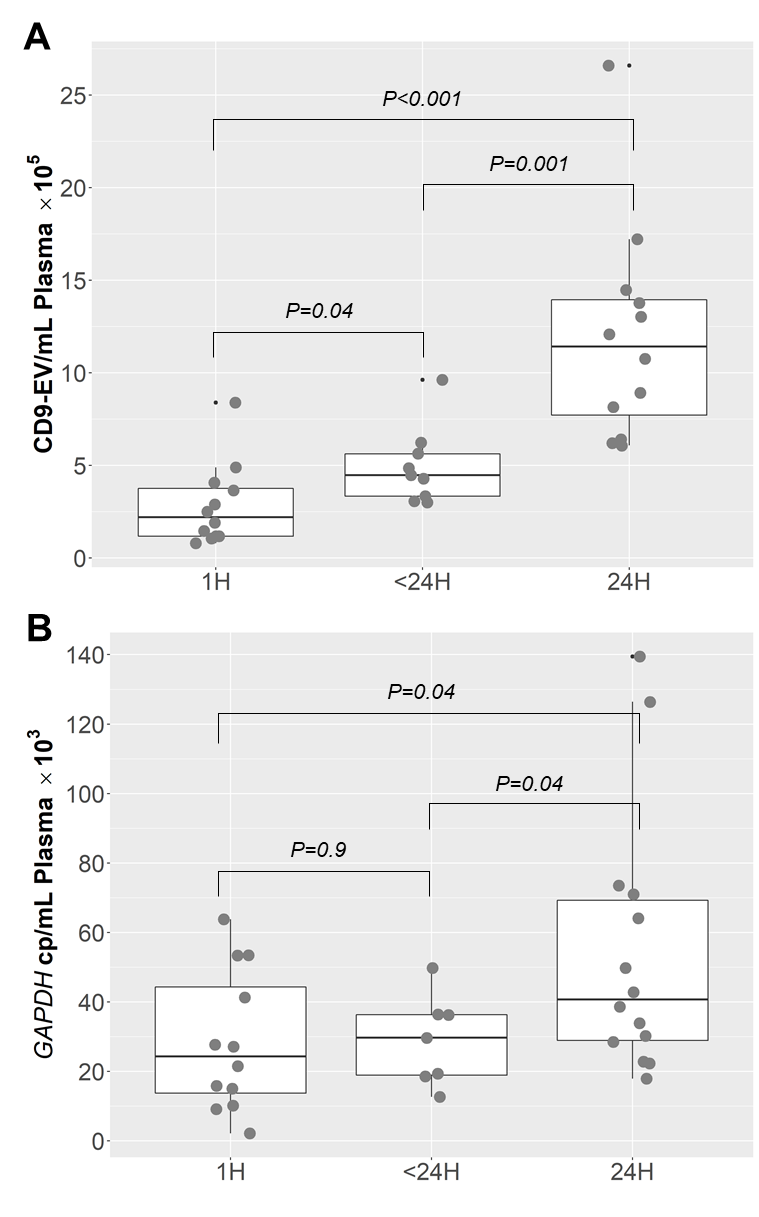
**

**Figure S2 - TRiFIC^TM^ Analysis of CD9-EV in plasma of cancer patients and *GAPDH* copies in their EV-RNA.** The boxplots shows in A) CD9-EV levels per mL plasma and in B) the *GAPDH* copies/mL plasma measured in EDTA tubes and processed at three time points (1 hour, within 24 hour and 24 hours) for 8 patients from each cohort 1 (at 1h and 24h) and cohort 2 (<24h). All measurements for CD9-EV were performed in duplicate.

**
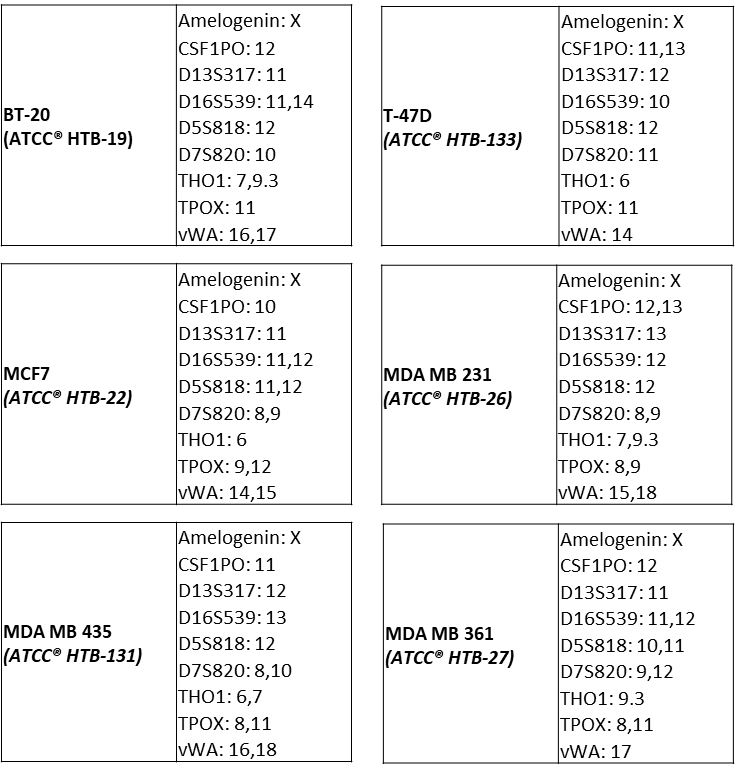
**

**Figure S3A:** Cell line STR Authentication measured by Powerplex 16 (Promega, cat: DC6531) of cell lines obtained from ATCC.

**
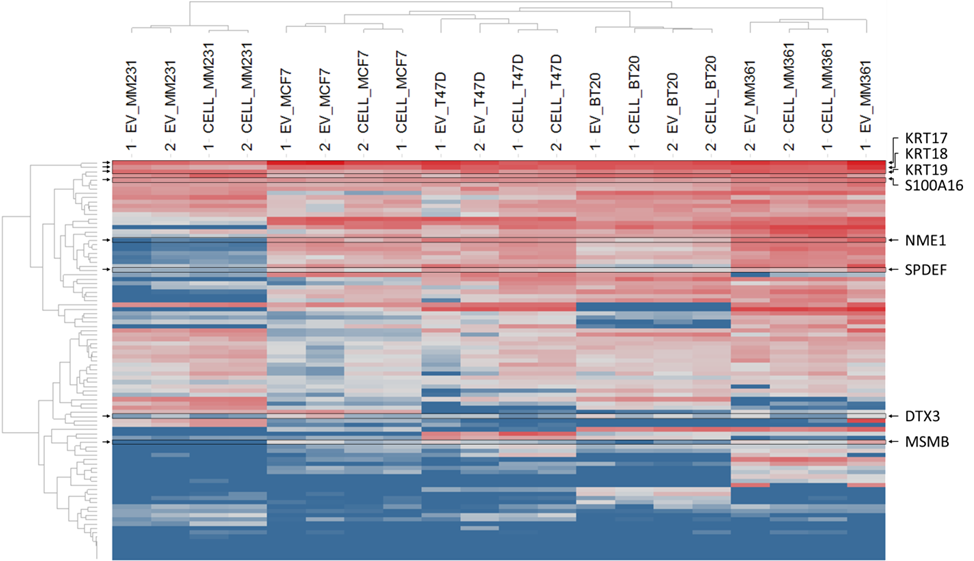
**

**Figure S3B - Expression profiling of breast cancer cell line mRNA and EV-RNA.** TaqMan Gene Expression Assays for 96 genes were used to evaluate the RNA expression levels by real time RT-PCR. The measurements were performed in duplicate on RNA from both cell line and EVs. Complete linkage cluster analysis was performed for both cell line mRNA and matched EV-RNA and demonstrated that EV expression profiles are similar to their parental cell line profiles. Each horizontal row represents a gene, and each vertical column corresponds to a sample with numbers indicating a separate analysis (1 and 2). Arrows at the right of the figure indicate the genes which are upregulated in EVs. Expression levels are colored at median (white), above median (red) or below median (blue).

**
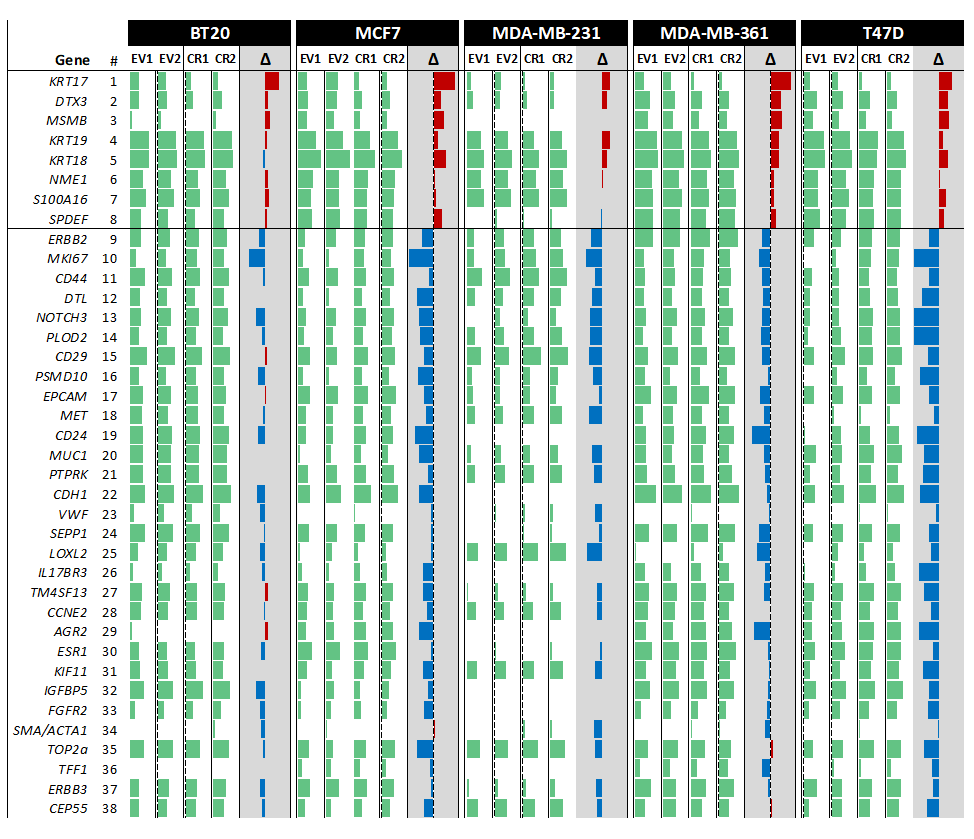
**

**Figure S4 - Differentially expressed genes in EV-RNA compared to their matched cellular mRNA.** The data are based on the independent duplicate analysis of 96 genes, but the figure shows only the 38 genes which overall were significantly different between EV-RNA and cellular RNA. Genes are indicated in the first column. The green bars for the individual cell lines represent expression levels of each gene relative to 3 reference genes (*HMBS*, *HPRT1* and *GUSB*) for two independent measurements of EV-RNA (EV1, EV2) and of cellular RNA (CR1, CR2). The difference in expression levels between EV-RNA and cellular RNA are shown by bars in red (EV>CR) or in blue (EV<CR). The first eight genes are more abundant in EV-RNA than in cellular RNA for almost all five cell lines.

**
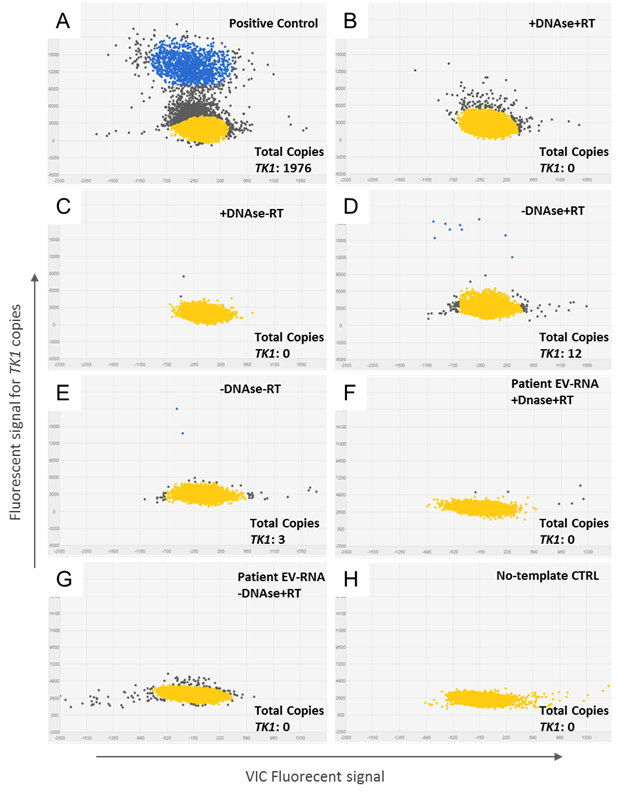
**

**Figure S5 - Analysis of an intronic region of *TK1* on cellular EV-RNA and patient EV-RNA.** The presence of DNA was evaluated in A) Celline DNA (positive control) and cellular EV-RNA treated in B) with DNAse and Reverse Transcriptase (RT); in C) with DNAse and without RT; in D) without DNAse and with RT; in E) without both DNAse and RT; and in patient EV-RNA treated in F) with DNAse and RT; in G) without DNAse and with RT; and H) show results for the no template control. No DNA was detected in both cellular and patient EV-RNA treated with DNAse. Blue: represent *TK1* copies, Yellow: represent empty wells, Grey: represent undetermined wells, VAF: Variant Allele Frequency.

**
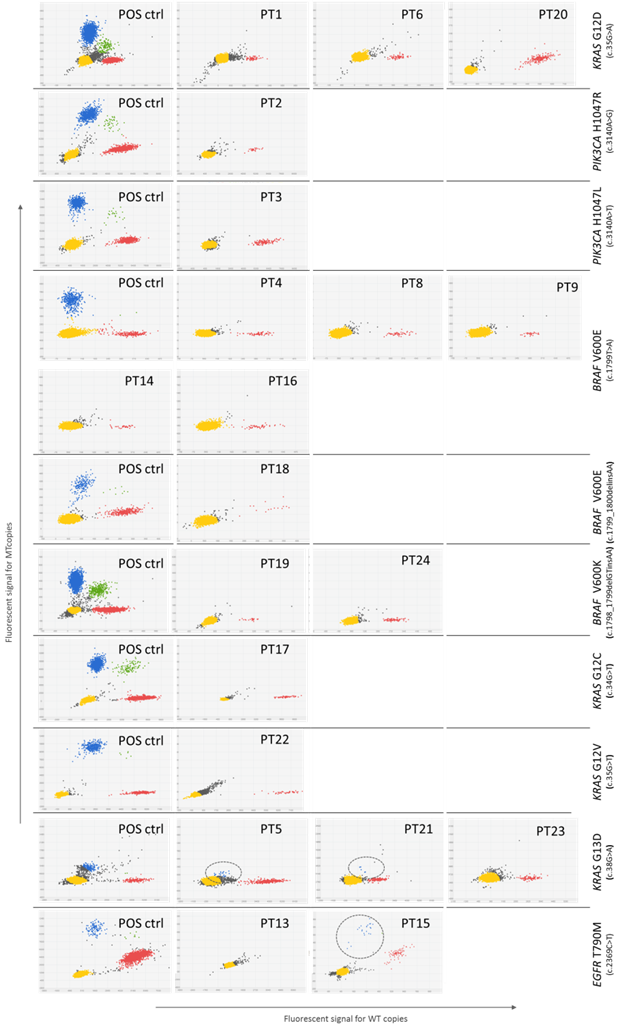
**

**Figure S6. Digital PCR dot-plots of target genes in 20 patients.** Digital PCR dotplots of target genes in 20 patients. The dPCR-plots for each of mutation assays are presented for cohort 1 of EDTA plasma at 1 hour (12 patients: # 1-16) and for cohort 2 of EDTA plasma within 24 hours (8 patients: #17-24). Dots represent wells with mutant copies (blue), wild-type copies (red), both wild-type and mutant copies (green), empty wells (yellow), and undetermined wells (grey). PT: Patient; POS ctrl: Positive Control.

**
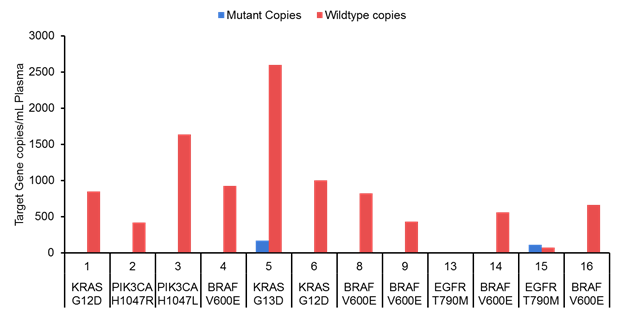
**

**Figure S7. Mutation analyses of target genes in EV-RNA of 12 patients derived from EDTA 24 hours plasma.** Wild-type and mutant copies/mL plasma measured by dPCR from EDTA plasma processed at 24 hours after blood draw from the cohort 1 cancer patients.
