# Supplementary material for: Detection of tumor-derived extracellular vesicles in plasma from patients with solid cancer
Source: BMC Cancer. 2021 Mar 24;21:315. doi: 10.1186/s12885-021-08007-z (PMC7992353; doi:10.1186/s12885-021-08007-z)
Supplement: Supplementary file 2 — Additional file 2: Table S1. Assays used for expression analyses and for wild-type and mutant copies of target genes. [file 12885_2021_8007_MOESM2_ESM.docx]

**Table S1. Assays used for expression analyses and for wild-type and mutant copies of target genes**

| **Gene** | **Type** | **Nucleotide change** | **Amino acid change** | **Assay ID** | **Manufacturer** |
| --- | --- | --- | --- | --- | --- |
| *PIK3CA* | Mutation | c.3140A>G | p.H1047R | AHPAVCD | Thermo Fisher |
| *PIK3CA* | Mutation | c.3140A>T | p.H1047L | AHLJ0TP | Thermo Fisher |
| *KRAS* | Mutation | c.35G>A | p.G12D | dHsaCP2500596 | Bio-Rad |
| *KRAS* | Mutation | - | Wildtype | dHsaCP2500597 | Bio-Rad |
| *KRAS* | Mutation | Screening Multiplex Assay | p.G12/G13 | 1863506 | Bio-Rad |
| *KRAS* | Mutation | c.34G>T | p.G12C | AN9HJKW | Thermo Fisher |
| *KRAS* | Mutation | c.35G>T | p.G12V | ANAAAYM | Thermo Fisher |
| *BRAF* | Mutation | c.1798_1799delGTinsAA | p.V600K | ANNKR4W | Thermo Fisher |
| *BRAF* | Mutation | c.1799_1800delinsAA | p.V600E | AN47WF2 | Thermo Fisher |
| *BRAF*  *(exon spanning)* | Mutation | c.1799T>A | p.V600E | ANCE3VC | Thermo Fisher |
| *BRAF* | Mutation | c.1799T>A | p.V600E | dHsaCP2000027 | Bio-Rad |
| *BRAF* | Mutation | - | Wildtype | dHsaCP2000028 | Bio-Rad |
| *EGFR* | Mutation | c.2369C>T | p.T790M | AHRSROS | Thermo Fisher |
| *PIK3CA* | Expression | - | - | Hs00907957_m1 | Thermo Fisher |
| *KRAS* | Expression | - | - | Hs00364284_g1 | Thermo Fisher |
| *BRAF* | Expression | - | - | Hs00269944_m1 | Thermo Fisher |
| *EGFR* | Expression | - | - | Hs00193306_m1 | Thermo Fisher |
| *GAPDH* | Expression | - | - | 4326317E | Thermo Fisher |
