# Supplementary material for: Detection of tumor-derived extracellular vesicles in plasma from patients with solid cancer
Source: BMC Cancer. 2021 Mar 24;21:315. doi: 10.1186/s12885-021-08007-z (PMC7992353; doi:10.1186/s12885-021-08007-z)
Supplement: Supplementary file 3 — Additional file 3: Table S2. Pearson correlation coefficient R based on expression levels of a 96 gene-panel. [file 12885_2021_8007_MOESM3_ESM.docx]

**Table S2. Pearson correlation coefficient R based on expression levels of a 96 gene-panel**

|  | MM231CELL1 | MM231EV1 | MM361CELL1 | MM361EV1 | T47DCELL1 | T47DEV1 | BT20CELL1 | BT20EV1 | MCF7CELL1 | MCF7EV1 |
| --- | --- | --- | --- | --- | --- | --- | --- | --- | --- | --- |
| MM231CELL2 | 0.996 | 0.964 | 0.245 | 0.295 | 0.309 | 0.295 | 0.485 | 0.464 | 0.422 | 0.409 |
| MM231EV2 | 0.977 | 0.980 | 0.288 | 0.358 | 0.357 | 0.362 | 0.503 | 0.493 | 0.476 | 0.487 |
| MM361CELL2 | 0.229 | 0.259 | 0.991 | 0.856 | 0.768 | 0.655 | 0.587 | 0.583 | 0.776 | 0.699 |
| MM361EV2 | 0.246 | 0.301 | 0.905 | 0.881 | 0.684 | 0.618 | 0.516 | 0.523 | 0.718 | 0.689 |
| T47DCELL2 | 0.347 | 0.370 | 0.763 | 0.667 | 0.993 | 0.855 | 0.662 | 0.641 | 0.800 | 0.721 |
| T47DEV2 | 0.340 | 0.405 | 0.703 | 0.673 | 0.930 | 0.911 | 0.619 | 0.618 | 0.782 | 0.781 |
| BT20CELL2 | 0.442 | 0.455 | 0.607 | 0.512 | 0.650 | 0.545 | 0.988 | 0.970 | 0.669 | 0.621 |
| BT20EV2 | 0.464 | 0.485 | 0.585 | 0.506 | 0.638 | 0.553 | 0.980 | 0.981 | 0.657 | 0.623 |
| MCF7CELL2 | 0.440 | 0.467 | 0.773 | 0.727 | 0.788 | 0.695 | 0.689 | 0.674 | 0.990 | 0.941 |
| MCF7EV2 | 0.362 | 0.429 | 0.674 | 0.707 | 0.702 | 0.758 | 0.595 | 0.607 | 0.912 | 0.953 |
| 1: first experiment,  2: second experiment | | | | | | | | | | |
