# Supplementary material for: Detection of tumor-derived extracellular vesicles in plasma from patients with solid cancer
Source: BMC Cancer. 2021 Mar 24;21:315. doi: 10.1186/s12885-021-08007-z (PMC7992353; doi:10.1186/s12885-021-08007-z)
Supplement: Supplementary file 4 — Additional file 4: Table S3. Differentially expressed genes in EV-RNA compared to cell line mRNA. [file 12885_2021_8007_MOESM4_ESM.docx]

**Table S3. Differentially expressed genes in EV-RNA compared to cell line mRNA**

| **#** | **Expression^a^** | **Gene** | **P-value** | **Per mutation P-Value** |
| --- | --- | --- | --- | --- |
|  | **Upregulated genes in EV-RNA** | | | |
| **1** | 8.33 | *KRT17* | 2.98E-05 | 0.0020 |
| **2** | 2.56 | *DTX3* | 0.0006 | 0.0020 |
| **3** | 2.78 | *MSMB* | 0.0047 | 0.0078 |
| **4** | 2.04 | *KRT19* | 0.0065 | 0.0039 |
| **5** | 2.38 | *KRT18* | 0.0073 | 0.0078 |
| **6** | 1.28 | *NME1* | 0.0074 | 0.0078 |
| **7** | 1.56 | *S100A16* | 0.0173 | 0.0117 |
| **8** | 1.75 | *SPDEF* | 0.0375 | 0.0410 |
|  | **Downregulated genes in EV-RNA** | | | |
| **9** | 0.27 | *ERBB2* | 9.45E-05 | 0.0020 |
| **10** | 0.06 | *MKI67* | 0.0004 | 0.0020 |
| **11** | 0.44 | *CD44* | 0.0010 | 0.0059 |
| **12** | 0.25 | *DTL* | 0.0011 | 0.0039 |
| **13** | 0.14 | *NOTCH3* | 0.0015 | 0.0020 |
| **14** | 0.18 | *PLOD2* | 0.0021 | 0.0020 |
| **15** | 0.33 | *CD29* | 0.0022 | 0.0078 |
| **16** | 0.24 | *PSMD10* | 0.0030 | 0.0039 |
| **17** | 0.43 | *EPCAM* | 0.0038 | 0.0117 |
| **18** | 0.40 | *MET* | 0.0039 | 0.0039 |
| **19** | 0.16 | *CD24* | 0.0042 | 0.0078 |
| **20** | 0.29 | *MUC1* | 0.0042 | 0.0020 |
| **21** | 0.38 | *PTPRK* | 0.0049 | 0.0039 |
| **22** | 0.30 | *CDH1* | 0.0073 | 0.0156 |
| **23** | 0.61 | *VWF* | 0.0092 | 0.0078 |
| **24** | 0.47 | *SEPP1* | 0.0093 | 0.0078 |
| **25** | 0.30 | *LOXL2* | 0.0096 | 0.0156 |
| **26** | 0.35 | *IL17BR3* | 0.0112 | 0.0078 |
| **27** | 0.39 | *TM4SF13* | 0.0118 | 0.0156 |
| **28** | 0.59 | *CCNE2* | 0.0121 | 0.0156 |
| **29** | 0.27 | *AGR2* | 0.0160 | 0.0313 |
| **30** | 0.65 | *ESR1* | 0.0168 | 0.0098 |
| **31** | 0.38 | *KIF11* | 0.0211 | 0.0195 |
| **32** | 0.49 | *IGFBP5* | 0.0230 | 0.0313 |
| **33** | 0.47 | *FGFR2* | 0.0230 | 0.0156 |
| **34** | 0.65 | *SMA/ACTA1* | 0.0307 | 0.0469 |
| **35** | 0.35 | *TOP2a* | 0.0307 | 0.0273 |
| **36** | 0.61 | *TFF1* | 0.0319 | 0.0625 |
| **37** | 0.55 | *ERBB3* | 0.0353 | 0.0430 |
| **38** | 0.46 | *CEP55* | 0.0402 | 0.0371 |
| ^a^ Gene expression in EV-RNA compared to matched cell line NA | | | | |
